# Public engagement and climate change: exploring the role of hairdressers as everyday influencers

**DOI:** 10.1057/s41599-026-06781-4

**Published:** 2026-02-26

**Authors:** Briony Latter, Sam Hampton, Denise Baden, Stephanie Hodgson

**Affiliations:** 1 Cardiff University, Cardiff, UK; 2 University of Oxford, Oxford, UK; 3 University of Bath, Bath, UK; 4 University of Southampton, Southampton, UK; 5 Utrecht University, Utrecht, The Netherlands

**Keywords:** Environmental studies, Geography, Psychology, Sociology, Business and management

## Abstract

Public engagement has a key role in the social transformations needed to address climate change, one form of which is climate conversations. This research focuses on a widespread and conversational space - hair salons. It engaged with sustainable salons across the United Kingdom and Republic of Ireland to explore these conversations in two studies. Thirty salon owners/directors were interviewed about hairdressers’ engagement with clients about climate change and sustainability (GoZero), and an intervention was conducted with 25 salons using eco-tips on mirrors to prompt sustainable hair care conversations (Mirror Talkers). The results show that hairdressers already have a strong understanding of public engagement, are able to ‘read’ clients and maintain trusting relationships. Climate and sustainability conversations are happening in sustainable salons and impacting clients’ mindset and behaviour, with the intervention viewed positively. This paper argues that hairdressers are a prime example of ‘everyday influencers’ on climate change, but their potential has not been fully realised.

## Introduction

The climate crisis demands substantial changes across society at multiple scales and by different actors, including governments, businesses, community groups and individuals (Hampton and Whitmarsh, 2023; Verfuerth et al., 2024; Westlake et al., 2024). Given the impact of climate change on people’s lives, public engagement forms an important part of responding to these changes. This can be through one-way forms of communication, such as information provision, or through two-way communication, which include structured formats such as climate assemblies or simply through conversations. Conversations can make climate change feel more relevant and create space for people to express concerns, hopes and emotions (Corner and Clarke, 2017; Ettinger and Painter, 2023). This can occur informally or in structured dialogues and dedicated spaces such as cafés and therapy rooms (Broad, 2024; Webster and Marshall, 2019).

In this interdisciplinary paper, we contribute to research on public engagement and climate dialogue by reporting results from two studies in hair salons: unique spaces in which conversations are key. Hairdressing is a widespread industry and is important economically and socially. Across the European Union, there are 1.7 million hairdressers and beauticians (Eurostat, 2020). In the UK, there are more than 61,000 hair and beauty businesses, largely small- and medium-sized enterprises (SMEs) (NHBF, 2023), contributing £5.1 billion to the economy and employing over 200,000 people (British Beauty Council and Oxford Economics, 2023). Hairdressers are renowned for building trusting and long-term relationships with their clients, undertaking emotional labour and acting as confidantes (Eayrs, 1993; Harness et al., 2021). Salons are important community spaces which can form and reflect social connections and (sometimes interconnecting) identities related to race, gender and class (e.g., Lawson, 1999; Lukate, 2022).

Research into the hairdresser-client relationship in relation to climate change and sustainability is very limited. However, Baden and Prasad (2016) found that hairdressers showed little awareness of salons’ environmental impact, and that most clients are affected by their hairdressers’ advice and would welcome tips on sustainable haircare. Recent research found that hairdressers are motivated to engage in conversations about climate change (Baden et al., 2024) and that clients think salons are appropriate places to have climate conversations (UHI Moray and Scottish Association of Marine Science, 2024). Here, we address the gap in research by investigating the role of hairdressers as everyday influencers on climate change and other environmental issues (e.g., water, waste, chemical use) in sustainable hair salons across the United Kingdom (UK) and the Republic of Ireland (ROI), specifically around engaging clients.

Empirical data is drawn from two studies (GoZero and Mirror Talkers) conducted from 2021 to 2024. These involved semi-structured interviews with salon owners/directors, and surveys and interviews with hairdressers, salon owners and clients following an innovative pilot intervention. The studies investigate the influence that hairdressers have with clients about climate change and sustainability. GoZero explored the research question: How do sustainable hair salons in the UK engage with clients about climate change and sustainability? Mirror Talkers addressed the research questions: (1) Do sustainability messages on salon mirrors prompt conversations between hairdressers and clients? (2) Do these messages reduce the environmental impact of clients’ hair routines?

This article makes several contributions to the literature on environmental communications. First, it demonstrates the unique skills and capacities of hairdressers to hold conversations and build trusting relationships with diverse publics, and to leverage these for sustainability and climate goals. Second, coining the term “everyday influencers”, it highlights the importance of decentralised public engagement, including the role of small businesses and public-facing professions for raising the profile of climate change and sustainability across society. Third, we highlight the need for business-related climate policy to expand beyond a focus on their direct emissions and other impacts, to leverage potential to yield influence.

## Literature review

In its broadest sense, public engagement covers education, awareness, training, participation, access to information, and international cooperation (Orr and Powell, 2023). It can prompt behaviour changes (Elstub et al., 2021) but also has broader value, for example, in influencing and creating informed support for climate policies (Cattino and Reckien, 2021). Public engagement also requires consideration of people’s values (Climate Outreach, 2024a) and who they trust – those who are viewed as “human, sincere, down to earth, kind, reliable [and] honest” (Climate Outreach, 2024b, p.10).

Dialogue-based approaches to public engagement shift from one-way communication to discussions. This allows diverse groups to collaboratively explore climate change across different contexts and purposes (Badullovich, 2023; Ettinger and Painter, 2023). Climate conversations are a key part of public engagement, in which it is important to identify common ground, seek connection over contest, and connect with current events (Nature Conservancy, 2023; Webster and Marshall, 2019). Ettinger et al. (2023) apply the healthcare concept of ‘teachable moments’ to climate communication, focusing on extreme weather events. They develop a dialogue-based framework to empower climate advocates and community organisations in facilitating discussions to enhance learning and inspire concrete actions. Climate conversations have also been used between scientists and local communities for *Curious Climate Tasmania* (Kelly et al., 2020) and for campaign outreach in the USA to overcome scepticism and create policy change using “deep canvassing”—a form of conversational outreach from the LGBTQIA movement focusing on listening in a safe and non-judgemental space (Yale Program on Climate Change Communication, 2022).

Climate conversations happen in many circumstances, though existing research mainly focuses on friends and family. Discussing climate change with close relations can increase knowledge, lead to more conversations and create a “proclimate social feedback loop” (Goldberg et al., 2019, p.14804). Yet although more than half of people globally say they *think* about climate change on a daily or weekly basis (58% for the UK; UNDP, 2024), only 26% of the UK public talk about climate change with friends or family at least once a week, and 13% never do (Ejaz et al., 2023).

There are differences in who holds these conversations, with younger people, more educated people, and active community members more likely to do so (Hampton and Whitmarsh, 2023). Also, a close relationship may not necessarily be positive in climate conversations. The risk of disagreement leads some people to avoid negatively impacting good relationships with others (Wright and Irwin, 2024). Research in the USA found that conversations about weather with family and friends rarely referred to pro-environmental action (Latkin et al., 2024), and conversations between climate activists and people they knew did not lead to action (Fine, 2024). However, an important factor to consider is pluralistic ignorance, defined as “inaccurate perceptions of others’ opinions”, as it can lead to people silencing themselves in climate conversations (Geiger and Swim, 2016, p.79) and has been shown to be a global problem, with people underestimating how willing others are to take climate action (Andre et al., 2024).

Multiple factors shape the low carbon choices of individuals, who can further influence others if supported in building their capacity to do so (Hampton and Whitmarsh, 2023). In the UK, high-impact low carbon behaviours by leaders, specifically politicians and celebrities, can positively influence people’s willingness to adopt the same behaviours (Westlake et al., 2024). People also conceptualise their ability and capacity to take climate action in different ways (such as collective, individual, limited or external), though more public engagement is needed for people to better imagine different ways to act—including actions that have a wider influence on those around them (Toivonen 2022).

In this paper, we focus on climate conversations in the unique setting of the hair salon. Most salons in the UK are SMEs, which have been underserved by climate policy and research (Hampton et al., 2022). Where this does address SMEs, it focuses on boosting resource efficiency, identifying barriers and drivers for implementing energy saving measures (Herce et al., 2024). However, there is increased attention on SMEs as pivotal actors for climate transformations, not just as consumers of energy and resources, but as enablers and influencers (Hampton et al., 2022). More broadly, in most businesses, over 70% of their greenhouse gas emissions are Scope 3, indirect emissions not produced by the company itself (UN Global Compact Network UK, n.d.). With regards to hairdressing, this includes the impacts of hair products sold or recommended to customers, as well as haircare practices outside the salon.

At the core of hair salons are hairdressers and the relationship they have with clients. This requires the ability to read body language and adapt to each client’s personality (Barbour, 2023). One study found that 68% of the British public trust hairdressers to tell the truth (Ipsos and Mumsnet, 2016), though for young people aged 11–16, this is much lower (19%; Ipsos, 2017). Their engagement can also vary depending on the length of their relationship (Garzaniti et al. 2011). Trust is key as they interact in a physically intimate space, and emotions often arise. Hairdressers sit in a “liminal space as listeners and counsellors” (Barbour, 2023, p.40), developing client relationships through informally playing the role of caregivers and friends, and providing emotional labour (Eayrs, 1993; Harness et al., 2021). Eayrs (1993, p.19) argues that “the practical yet intimate nature of hairdressing provides an exceptional way to observe trust”. This role has been formalised in hairdresser training, for example, in mental health (de Vergès, 2024) and domestic violence (McCann and Myers, 2023).

Hair salons have a strong connection to their local area. They are places that people repeatedly visit, with most people in Britain visiting their hairdresser every one or two months and interacting with the same hairdresser (YouGov, 2012). During the Covid-19 pandemic, research in Australia found that salon closures were keenly felt as a loss of social connection and a space away from everyday life (McCann, 2024). Salons could be seen as a “third place”, a term first coined by Oldenburg (1989, p.293) to refer to “public places that host the regular, voluntary, informal, and happily anticipated gatherings of individuals beyond the realms of home and work”.

Gender is also an important consideration in the context of businesses and climate action. Most hairdressers and beauticians in the European Union are women and are under 50 years old (Eurostat, 2020), and more than 80% of business owners in the personal care industry in Britain are women (British Beauty Council and Oxford Economics, 2023). Global research into SMEs found that more women than men had changed their business practices to address climate change but needed more information before doing so (World Trade Organization, 2022).

Almost no research exists which explores the relationship between hairdressers and clients through a climate or sustainability lens. While there is an example of experimental work in Australia exploring climate conversations with a non-professional hairdresser, it was one aspect of a wider event and only two people took part (Hamilton et al., 2021). However, a salon in Australia has run workshops with hairdressers to help them engage in climate conversations with clients (Readfearn, 2023) and a small multidisciplinary student project in Scotland piloted climate conversations in salons, finding that clients thought salons were appropriate places to have these conversations and that clients also spoke to other people (such as friends and family) afterwards about their experience (UHI Moray and Scottish Association of Marine Science 2024).

Also, in the UK, Baden (Baden et al., 2024; Baden and Prasad, 2016; University of Southampton, 2019) has pioneered impact-focused research in this area, launching a sustainable salon certificate and researching salons’ environmental impacts. Salons consume significant quantities of energy and chemicals and produce substantial (often non-recyclable) waste (Phorest, 2019). However, a survey of 776 salon clients found that they thought their hair appointments were more sustainable after their hairdressers had attended a sustainable salon event and were more willing to consider sustainability issues in their own haircare practices (Baden and Prasad, 2016). A study of hairdressers’ motivations to adopt sustainable hair practices revealed that, in addition to cost savings and reputation, many believed advising clients on sustainable haircare enables them to be seen as professionals, raising the status of their occupation (Baden et al., 2024). Broader research found that SMEs, particularly hairdressers and people working in construction trades, have a key role in influencing others around decarbonisation (Eadson et al., 2024).

## Methods

This paper brings together data from two research projects. GoZero (2022-2024) investigated governance arrangements for SME decarbonisation around the UK, selecting five sectors and five places for empirical data collection (Hampton et al., 2024). Hairdressing was selected due to its scale, energy intensity, and unique influence potential. Mirror Talkers (2021–2022) was a 12-month initiative led by the Eco Hair and Beauty project in collaboration with the Green Salon Collective (GSC). It involved an intervention to test whether tips on sustainable hair practices on salon mirrors could prompt conversations between hairdressers and clients about these practices and reduce the impact of their hair routines. Due to the focus on sustainable haircare and the impact of broader issues such as water and waste in salons, we explore both sustainability and climate change across the projects.

### GoZero

We identified UK salons that were already engaged with climate change and/or sustainability. This was mostly through membership of the GSC, with a small number recommended by an existing contact in the sector.

Our sampling strategy aimed to capture a broad range of sustainable salons across the UK. We aimed for geographical diversity, contacting salons from all regions of the UK. We also sought a mix of salon sizes, from established SMEs operating at multiple sites and employing a team, to small independent operations, and those providing co-working spaces. Standalone barbershops were excluded as very few are part of the GSC and they have a different social function to hair salons (Barber, 2008). Most salons catered primarily to women, although many offer unisex services.

**Fig. 1:**
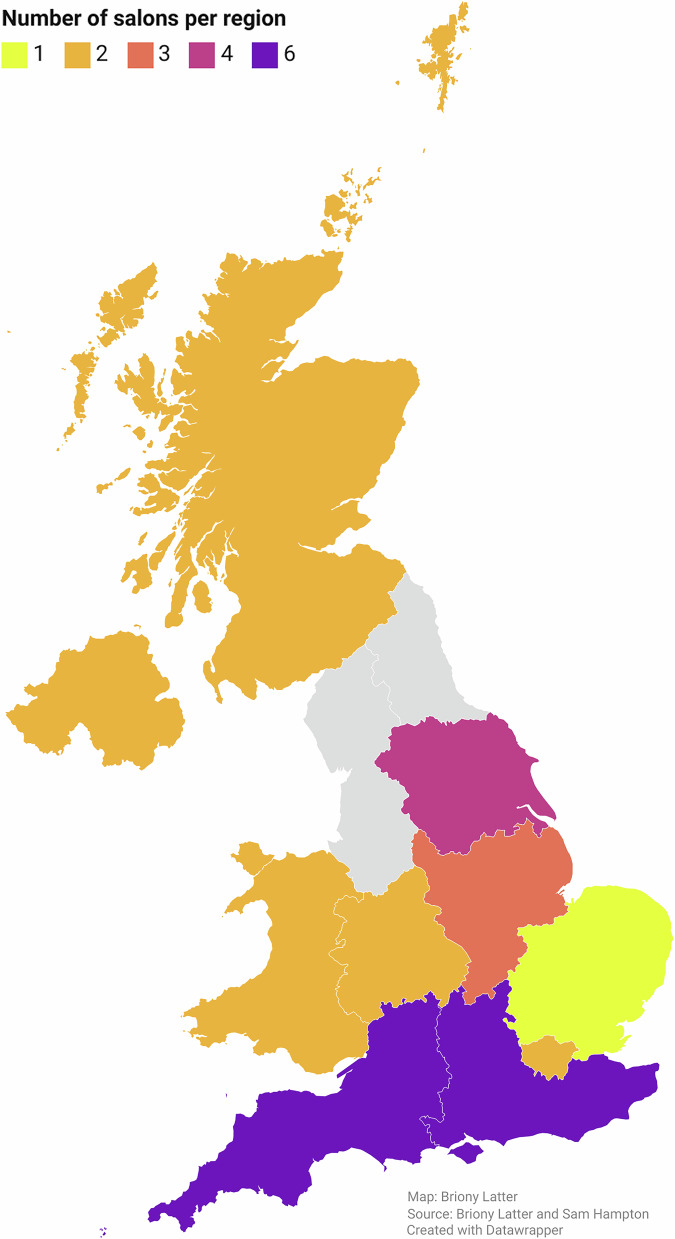
GoZero: Number of salons interviewed per region (UK).

We contacted 190 salons via email, resulting in interviews with 30 salons, represented by owners or directors capable of discussing business operations and client engagement. Of these, 27 were members of the GSC, and three were not. Seven salons specialise in curly and/or textured hair; five cater to Afro hair. Regarding business structure, 22 operate with a team of hairdressers, four are co-working spaces, and four are one-person businesses. The sample comprises 24 salons in England, two in Northern Ireland, two in Scotland, and two in Wales (Fig. 1).

Semi-structured interviews were conducted using online video calls and lasted 25–50 min. Participants provided information about their salon’s sustainability and climate actions then their engagement with clients on these topics, including the content of the conversations, influence and trust, and wider influence. For example, questions included “Do you engage with your clients about the climate change and sustainability actions that your salon is taking?”, “Do you have any examples of when you felt you’ve been able to influence clients on climate change or sustainability?” and “How much do you think clients trust you (in your role as a hairdresser) to talk about climate change and sustainability?”. See the supplementary material for the full interview protocol.

Interviews were audio recorded and transcribed, with participants providing informed consent to take part. Participant data is presented anonymously, numbering participants from one to 30 (for example, hair salon owner/director 1 = H1). Interviews took place between June and July 2024. Data were analysed in NVivo using inductive thematic analysis, ‘coding’ text to draw out common meanings and gradually developing these into broader themes (Fugard and Potts, 2019).

### Mirror talkers

**Fig. 2:**
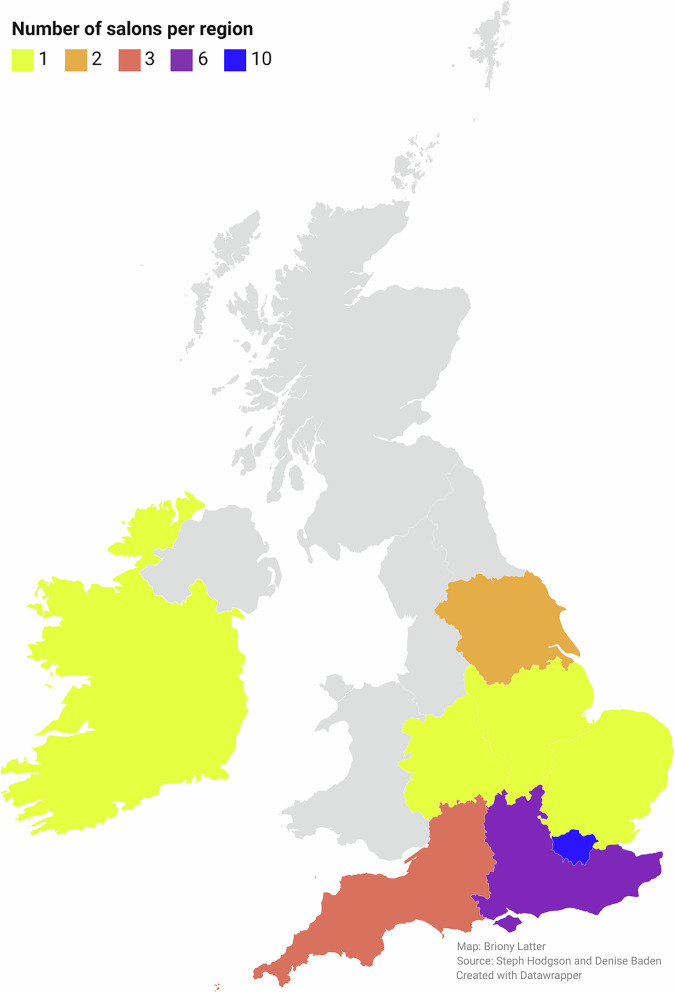
Mirror Talkers: Number of salons involved per region (UK and ROI).

Hair salons with sustainable business practices, including GSC members and those who obtained the sustainable salon certificate through Eco Hair and Beauty, were invited to take part via newsletters and other communication channels. Twenty-five salons took part (19 businesses—some owned multiple salons); 24 were based in England and one in the Republic of Ireland (Fig. 2).

**Fig. 3:**
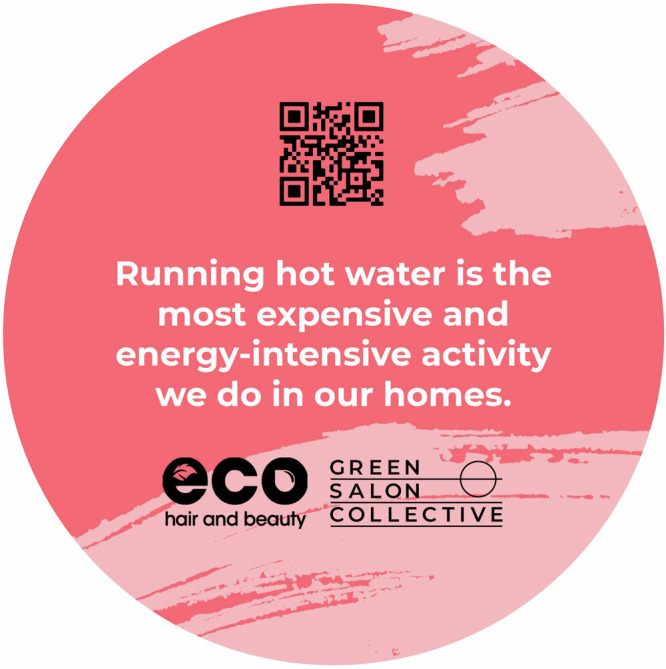
Example of a Mirror Talkers sticker.

The intervention provided salons with materials and training prior to data collection. There were 12 sticker variations, with facts, questions, “true or false” statements and a QR code for further information (Fig. 3; see supplementary material for all designs). Salon owners/directors chose their preferred selection of stickers; the amount they were sent corresponded to the number of chairs per business (272 stickers sent in total). They were also sent written and video educational materials and additional stickers explaining the study. Some took part in optional training sessions from the research team, with further information about the Mirror Talkers and an opportunity to ask questions. Salons displayed the Mirror Talkers for 2–6 months and had conversations with clients about sustainable hair care at appropriate moments.

There were two methods of data collection, for which participants provided informed consent. Firstly, online Google Forms surveys were provided to hairdressers, salon owners and clients which could be accessed at any time. Clients were encouraged to complete this as soon as possible after their conversations, and hairdressers and owners completed this after approximately two months of having the Mirror Talkers. From the 25 salons that took part, 97 responses were received from clients (*N* = 59), hairdressers (*N* = 30) and salon owners (*N* = 8). Surveys took on average five minutes to complete. The surveys included questions about training materials, engagement with the Mirror Talkers, and client sustainable haircare intentions. For example, “How effective were the Mirror Talkers at prompting conversations around sustainable haircare practices?” and “Do you believe those conversations will have a positive effect on your clients’ haircare routines?”. Data was collected between March and September 2022.

Secondly, semi-structured interviews were conducted with seven participants: six salon owners and one client; all opted to give interviews after completing a survey. These lasted 10–20 min. The interview guide balanced closed and open questions to suit time-limited participants. Salon owners were asked about why they chose particular Mirror Talkers, client conversations, and changes to the Mirror Talkers and training. The client was asked about their conversation with the hairdresser and sustainable haircare intentions. For example, questions included “How did your conversation with your hairdresser/client on this topic unfold? What did that interaction look like?” and “Did you scan the QR code on the Mirror Talker?”. Interviews were conducted in July 2022 and were audio recorded and transcribed. See the supplementary material for the full list of survey questions and the interview protocol.

Salons were identified but participants were not named and comments were not linked to specific salons. Analysis was undertaken by one researcher and checked by the second. Survey data was analysed in Microsoft Excel using descriptive statistics for nominal data directly answering the research questions, while the remaining categorical responses were summarised using frequency and percentage counts. Interview data was analysed using Reflexive Thematic Analysis (Braun and Clark, 2019), following an inductive approach to identify recurring themes.

## Results

### GoZero

GoZero aimed to explore how sustainable hair salons in the UK engage with clients about climate change and sustainability. Firstly, salon actions around sustainability were important to identify upfront to understand their level of engagement with these issues and the context in which client interactions took place. The interviews with salon owners/directors showed that sustainable hair salons across the UK are taking actions to reduce their environmental impact. This mainly related to waste and recycling, particularly hair and metals. More than half spoke about sustainable or ethical hair products, water, energy, lighting, towels and gowns, recycling contaminated hair foils and reducing chemical use. Plastics and non-hair products such as toilet roll and cleaning products were also mentioned.

There are four key themes relating to clients: (1) The social fabric of salons; (2) Client conversations and “reading” clients; (3) Physical items prompt conversations; (4) Influencing client behaviour and mindset.

**Theme 1: The social fabric of salons**

The social fabric of hair salons relates to trust, client relationships and how salons are a social space. This theme refers to general salon characteristics and client engagement as well as more specific findings about climate change and sustainability. Most participants (21) spoke about how salons have characteristics which make them unusual or influential spaces. One aspect is the continued relationship hairdressers have with clients over time, often over many years, with one stating that for long term clients, “you know their life” (H24).

*“Hairdressers are very good at weaving in narrative. If they think it’s important, they can influence their client for sustained periods of time. We’re not talking about one time, we’re talking about years of sustained influence.”* **- H2**

*“We have situations […] where we have at least three generations of family, if not four. So our level of influence generationally, as well as demographically, is huge.”* **- H30**

Seventeen participants explained how clients trust them because of their relationship or because it is simply part of their role: “Our job is to gain trust, isn’t it?” (H3). One said “it’s easy to plant a seed” (H17) because the client trusts them. Two mentioned that salons are spaces where clients are relaxed, happy and away from other commitments, therefore “you can influence people quite easily” (H10). Others described it as a space where clients feel safe discussing anything, and one mentioned that salons were missed during the Covid-19 lockdown. Some also discussed how a hair salon is “quite an intimate space physically” (H21) and therefore “you have to have the ability to make somebody feel at ease straight away” (H20), with hairdressers being seen as a “confidante” or a “counsellor”. However, one mentioned how this role can leave hairdressers feeling “a bit heavier than when they arrived. […] You’re sort of letting people dump a little bit more onto you than the other way around” (H7).

*“Clients trust their hairdressers, otherwise they wouldn’t keep coming back to us. It’s an opportunity for them to air their feelings and their opinions in a safe space.”* **- H28**

The relationship between hairdressers and clients extended to climate change and sustainability. 28/30 thought their clients trusted them to talk about these topics. Some linked this to general trust because “when you have a hairdresser, you have to trust them anyway. No matter if it’s about sustainability or anything” (H4). Four explained that trust with long-term clients was particularly strong because “we’ve built that rapport beforehand” (H18) though one felt engagement might be harder if climate and sustainability have not previously been conversation topics. Compared with other client-service relationships, salon appointments were seen to be longer, helping to build a “close relationship” (H12).

*“An actual hour sat with one person having a conversation - there’s not many other industries that do that.”* **- H10**

*“You can do [your food shop] without speaking to a single person. We’re one of the few service providers that […] have that amount of verbal communication with a person face to face, you know? And that’s hugely influential because it’s relatively uncommon now.”* **- H12**

Interviewees explained that client trust on sustainability is also built by salons taking visible action, communicating their efforts, and personal commitment from owners:

*“I think of all the years where all that foil went to landfill. […] [Clients] trust me because I can go on about it. […] And I did loads of research. And I felt really bad.”* **- H27**

*“Once you’re confident in your knowledge, people become more trusting in you and can easily be influenced to do what you’re saying if it benefits them.”* **- H3**

**Theme 2: Client conversations and ‘reading’ clients**

Similarly to Theme 1 (the social fabric of salons), this theme shows how salons engage with clients about climate change and sustainability but also how they engage with clients generally. Most (24) salon owners/directors spoke about their ability to “read” clients and tailor engagement accordingly, developed through years of experience. This includes quickly understanding clients’ attitudes and interests as they are “always trying to find common ground” (H3), picking up clues from clients’ clothing, interests and how they act.

*“It’s our job to read the clients. […] You can usually tell [by] how they dress, what they’re wearing. That sounds odd, but everyone has got a way about them, really. Years of experience tell me I’ll never have a consultation with someone I’ve never met when they’re sitting there with a gown on. I want to see what they’re wearing and what type of person they are.”* **- H14**

*“I’ve been in the industry nearly 30 years so I can usually gauge and work out what the client wants by just their mannerisms. […] When a new client comes through the door, you’ll fish. You’ll drop little things in to see what picks up. […] From the consultation all the way through, you’ll be fishing for a common denominator.”* **- H19**

*“I don’t want to be mean, but yeah, like if someone’s got an allotment, they’re going to be interested [in climate or sustainability].”* **- H9**

The climate and sustainability topics discussed with clients tended to relate to pro-environmental actions the salon is taking. Most (23) said these conversations arise from both the client and hairdresser, mainly relating to hair recycling, hair products, refills, chemicals, foil recycling and waste. Two mentioned that unusual topics, such as hair recycling, are easier to talk about.

A large majority (25) said they engage with clients about climate and sustainability topics that are *not* related to hairdressing. Transport was mentioned by several participants, mainly electric cars, but two mentioned flying, public transport and electric scooters. Other topics included material consumption, food and drink, climate change as a generational issue, plants, and politics. Extreme weather and recent news also prompted conversations.

*“People like to talk about the weather. So we talk about ‘Well, it’s all to do with climate change, isn’t it?’ […] We have those conversations quite often.”* **- H4**

Nineteen said clients respond positively to climate and sustainability conversations, particularly regarding salon actions, with one noting how people can be “surprised and shocked” (H29) at the impact of their hair appointment. While several mentioned a lack of engagement from some clients, this rarely included negative responses. Eleven also spoke about perceptions of climate change and sustainability, for example, they accepted that “not everyone is bothered” (H10), with two mentioning a lack of client understanding of what an “eco” salon means.

*“We’re taught to understand body language quite well and if they’re doing a closed answer to your question, then it’s time to move on.”* **- H14**

While a few salon owners/directors were happy to speak to everyone about climate change and sustainability, many experienced differences in clients’ engagement or could tell through prior experience who would be interested. As one participant put it, “we will talk to the *right* clients about it” (H19). Age was a common differentiator, but opinions differed on which generations were more engaged. One participant whose clients tend to be an older generation felt they are “quite open to the conversation” (H26) and another thought their older clients are better at recycling than younger clients because of their ‘make do and mend’ attitude. Conversely, others felt that middle-aged or older clients were more difficult to engage with:

*“Kids are easy. Mums are easy. Teenagers, if you can get them chatting are easy. Retired, as I said, they have to see it on The One Show [UK television show].”* **- H9**

*“The older clientele are a bit more difficult. They’re kind of set in their ways. And the younger clientele, they’re not ready for that. So you’re sort of looking, the kind of 25 to 45 mark.”* **- H11**

Yet, many experienced how it was down to individuals or would pick up on whether clients had certain interests that could start conversations, for example, product purchases or a client’s personal interests.

Some were more comfortable discussing climate change and sustainability or being forthright about their opinion than others. For example, some felt they should have conversations “from a no judgement point of view” (H30) or “say it in a way where people don’t feel embarrassed or pressured” (H4). Whereas others felt that “sometimes you have to be honest and frank” (H15) or they “actually quite enjoy challenging people” (H8). Climate change was seen as political by some, with two saying it is “a grey area” for conversation. Linked to this, there were differences in whether they saw politics as an acceptable conversation topic, with one saying it is a “big faux pas” (H28) in hairdressing, yet two mentioned recent client discussions about political parties and another said acceptable conversation topics have shifted over the years:

*“There always used to be a rule in hair and beauty when you did your training that you didn’t talk about […] politics, religion and sexual orientation […] a couple of decades ago. And that has changed.”* **- H30**

**Theme 3: Physical items prompt conversations**

This theme demonstrates a different way in which salons engage with clients; most participants (19) spoke about how physical items in their salon prompt engagement with climate change or sustainability. This included stickers which were seen as conversation starters—“[they] get people chatting” (H9). The GSC have stickers available to purchase, including the Mirror Talkers (available for anyone after the intervention), which include information about recycling, waste and a ‘green fee’ that some salons charge.

Some salons display posters (about palm oil, recycling and general sustainability actions their salon is taking), brand-provided materials (such as magazines) or have the ‘Green Bible’ (GSC’s sustainability guide) available to read.

*“We use those Mirror Talkers […] and they’re really good cause […] it will help spark conversations.”* **- H4**

*“People have tapped the door before and said ‘oh, what’s this?’ […] It’s the Green Salon Collective stickers that’s doing that. So they are creating awareness.”* **- H13**

*“You will have people that have seen what’s on the back of the toilet door [poster] and then say, ‘I didn’t realise how much you did’ and talk about it.”* **- H29**

Other conversation prompts include unconventional towels or gowns, eco heads on showers, recycling bins, product refill stations and sustainability awards won by the salon. Some even have boxes of hair for clients to take home for composting. It was felt that props helped with climate and sustainability conversations because these topics are “not always something that they [clients] would naturally bring up if the prompts weren’t there” (H11), meaning that “it’s relevant because it’s part of the salon” (H13). Non-hair related items such as sustainable toilet roll, salon lighting and plants also prompt conversations.

**Theme 4: Influencing client behaviour and mindset**

Most salon owners/directors (24) said they were already using, or could use, their influence with clients for climate and sustainability engagement. Many also said their influence is not being used to its full potential:

*“Hairdressers are massively underrepresented from an influence perspective. We can convince you to basically do anything that we ask you to.”* **- H2**

*“We can influence people quite easily in conversations and we can lead conversations a certain way. […] As an industry we could be a lot better at that.”* **- H10**

Some even felt that hairdressers have *responsibility* to use their influence for good:

“*You should use that platform for a better cause and not just talk about crap*.” **-H8**

The most direct form this takes is related to hair, particularly the consumption of haircare products. One participant said almost all clients now use their refill station and another hoped they are “changing the behaviour to some extent, even if it is just directly related to what they’re using on their hair” (H23). There were also many instances of participants influencing clients’ sustainable practices and considerations at home or in clients’ businesses. This included water and plastic use, recycling, taking hair or towels home to reuse, drinks, toilet roll and cleaning products. Salon owners/directors explained how salon practices and purchases can create change, for example, using bamboo toilet roll and having milk delivered in glass bottles led to clients taking up these practices at home. Conversations also prompted this, such as making clients consider how much plastic they have in their bathroom or using cooler water during showers.

Instances where participants have been able to influence clients on climate change or sustainability topics that are *not* related to hair or the salon were rarer. However, one mentioned influencing clients’ banking choices, and two mentioned food and diet:

*“None of [my banking] contributes to oil tycoons or anything like that […] About 20%, so far, of my clientele have left their banking because I’ve just said ‘do you realise that this happens?’ and they’re like ‘oh my God, no’.”* **- H8**

*“A couple of my clients have taken recipes off me. That would probably be my main thing with this meat free [influence].”* **- H11**

While few felt they do not influence clients on topics other than haircare, uncertainty was more common, with ten being unsure about their influence: “I couldn’t tell you if I have, but I’m hoping just by talking about it, that’s doing something, isn’t it?” (H14). This uncertainty was for varying reasons, such as already having likeminded clients or being unsure whether clients truly absorb, and act on, the information.

Education and knowledge were spoken about by almost all participants (28). This partly related to the wider influence hairdressers can have towards other businesses, the hairdressing industry and brands. However, it also included how climate and sustainability actions tend to be led by a knowledgeable owner who then influences their hairdressers and clients: “Once your team buys into it, your clients will” (H16). Some had undergone training or gained knowledge through other means and were creating educational resources themselves or sharing knowledge with other salons. Yet, a high interest in climate and sustainability was seen by a couple as being an “eco warrior” and one mentioned how “people still think you’re a little bit odd […] They think I’m all Greta Thunberg and some kind of activist” (H6) for wanting to make their salon sustainable. Another owner thought that discussions about climate action can be “a bit confrontational […] it’s like you’re either all for it or you don’t know anything about it […] almost like it’s cool to have a strong belief” (H3), with them suggesting an inclusive and collaborative approach with clients is needed. There were mixed experiences in educating their hairdressers, with some teams educated on these issues and understanding the salon’s values, and a lack of engagement or knowledge at other salons which in some cases was felt to be related to the young age of some hairdressers.

### Mirror Talkers

Mirror Talkers aimed to explore whether sustainability messages on salon mirrors prompt conversations between hairdressers and clients, and if these messages reduce the impact of clients’ hair routines.

### Survey results

Table 1 presents descriptive statistics for the survey questions that directly addressed the research questions and for which nominal responses were given. Hairdressers and salon owners rated how effective the Mirror Talkers were at prompting conversations around haircare practices (Research Question 1). Hairdressers gave an average rating of 3.36 (SD = 1.06), while owners gave an average rating of 3.25 (SD = 1.28).

**Table 1:** Mirror Talkers survey questions directly addressing the research questions.

	Research question 1: “How effective were the Mirror Talkers at prompting conversations around sustainable haircare practices?“		Research question 2: “Based on this experience, how likely is it that you will change your haircare routine?“
	Hairdressers	Owners	Clients
Count	28	8	53
Mean	3.36	3.25	4.15
Standard Deviation	1.06	1.28	0.89

Five-point Likert scales were used from 1 (“Not at all”) to 5 (“Very effective”) for research question 1 and from 1 (“Not at all”) to 5 (“Very likely”) for research question 2.

**Fig. 4:**
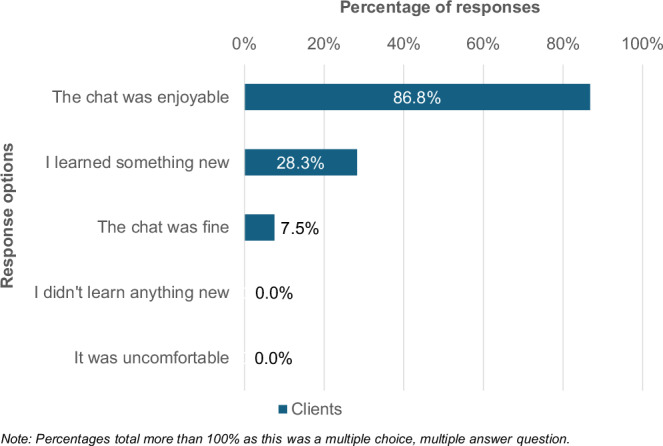
Did you discuss the Mirror Talker with your hairdresser? If “yes”, tell me how that went. Select all that apply. (*N* = 53).

When asked more generally about the conversations prompted by the Mirror Talker stickers, the most popular responses from hairdressers and owners (37 responses) were “I was confident” (51.4%), “they were fine” (45.9%) and “they were enjoyable” (21.6%). Only 5.4% said they were not confident. From a client perspective (Figs. 4), 86.8% found the conversations enjoyable and 28.3% learnt something new. When clients were asked why they scanned the QR code on the sticker (59 responses), 64% said they were prompted to by their hairdresser and 32.2% were curious or wanted to learn more.

**Fig. 5:**
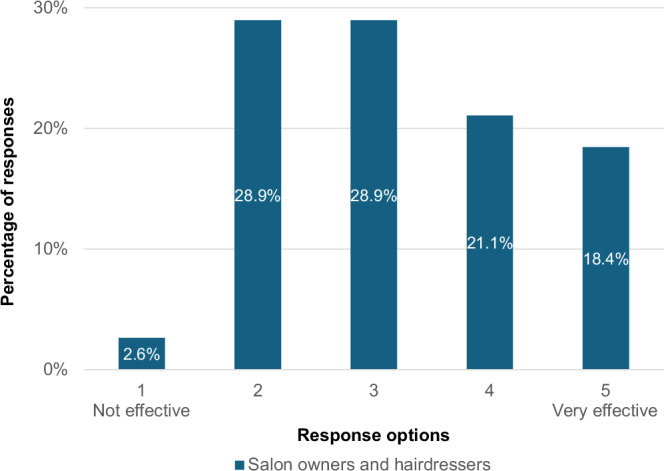
How effective were the Mirror Talkers at prompting conversations around sustainable haircare practices? (*N* = 38).

Hairdressers and owners were asked how effective they felt the Mirror Talkers were at prompting conversations around sustainable haircare practices (Fig. 5). While only one said they were not effective, the responses were mixed and this was reflected in open text box explanations from owners. For example, one mentioned how “it was hugely dependent upon the hairdressers and receptionists/assistants bringing up the conversation with their client” yet others said many clients asked about it and were “very interested in the information” as well as being “keen to learn and wanted to act on it”. This is also illustrated by an owner who said: “some clients thought the information was brilliant and then we had the negative Nellys”.

**Fig. 6:**
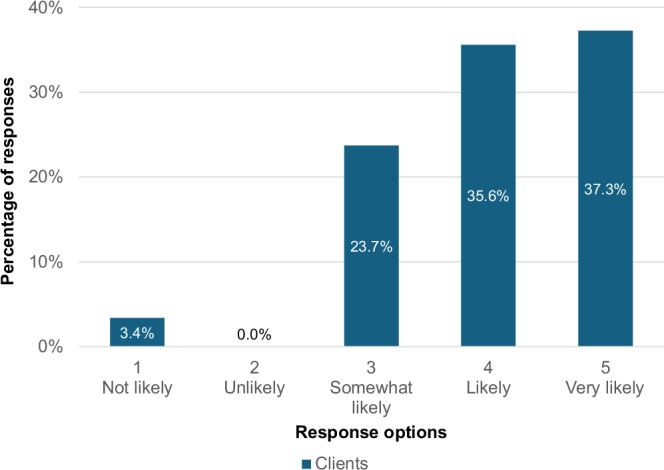
Based on this experience, how likely is it that you will change your haircare routine? (*N* = 59).

To address research question 2, clients were asked to rate the likelihood of changing their haircare routines following the experience. The mean response was 4.15 (SD = 0.89; see Table 1). While clients were the only group to receive a Likert-scale question directly linked to potential behaviour change (allowing for descriptive statistical reporting), hairdressers and owners responded to a categorical question about whether they believed the conversations would positively influence client routines. Among those who responded, around two thirds (67.9%) of hairdressers and 57.1% of owners felt the conversations would have a positive impact on their clients’ haircare routines (35 responses). One owner described the conversations as “a small seed planted in the client’s head” and that in their experience, “clients do come back and start the convo up again and explain how they have made changes at home because of us”. The conversations were seen to be a “layer” of environmental information for clients, “rather than be[ing] the sole catalyst for change”. However, 21.4% of hairdressers and 14.2% of owners said they were “on the fence”—indicating a neutral or mixed view—about whether the conversations could have a positive impact, and 42.9% of owners said they did not know. Yet client responses were mostly positive; 72.9% said they were very likely or likely to change their haircare routine after the conversation (Fig. 6).

### Interview results

The interviews also helped to answer both research questions, mainly exploring client conversations in greater detail. Generally, salon owners felt the Mirror Talkers and client conversations were positive. The client felt similarly because the Mirror Talker prompted different conversations to the norm, and they saw hair salons as a particularly suitable space for sustainability conversations with a “captive audience”. The tone of the conversations was also felt to have an impact on subsequent behaviour change.

*“You don’t feel like somebody is lecturing you. […] Talking about sustainability, talking about something in a really nice, warm, cuddly, informal environment is a brilliant way of starting to […] get people thinking about it.”* **- Client**

Six out of seven interviewees thought the stickers were most effective when the content felt relevant—particularly hot water and energy use—but could also be linked to topics outside of the salon, with one interviewee explaining how the energy price increases in 2022 meant it was a relevant and timely conversation to have.

*“It was [a] really on-topic conversation to be able to have, to bring all the eco stuff in, because people wanted to save money*.*”* - **H1 Mirror Talkers**

Three said that Mirror Talkers which included questions or mentioned a personal gain (such as cheaper water and energy bills) prompted more conversations. There was some evidence that the Mirror Talkers prompted behaviour change, with four participants reporting increased sales of more sustainable products (dry shampoo, shampoo bars, leave-in conditioner). One participant also felt the Mirror Talkers supported more sustainable water and energy practices in their salon. All interviewees said the stickers need to be accompanied by concise and easy to understand support material for hairdressers to take on board and share with clients.

Habits were also spoken about by three salon owners. This related to how clients’ haircare practices become habitual even when alternatives would be more sustainable or better for their hair. One also saw sustainability as needing to become habitual for hairdressers, and another explained how training could increase hairdressers’ engagement with clients on the topic.

*“Anyone needs reminding, constant reminding, for it [sustainability] to become a habit. […] It’s like anything in the salon, we’re constantly reminding the team to do things.”* ***-*** **H6 Mirror Talkers**

*“I struggled to get my team to talk to clients […] about something perhaps that they don’t feel they are confident in. […] So there could be a training issue there****.” -*** **H3 Mirror Talkers**

For the six owners, the training element was important as they felt it could make their hairdressers more confident, in turn positively impacting how effective the Mirror Talkers were. One owner said the training motivated their hairdressers to have sustainable haircare conversations with clients, and the client felt that Mirror Talkers are “a really good opportunity” with the right information and training in place.

## Discussion

This paper brings together two research studies with the aim of exploring the influence that hairdressers—as widespread professionals working in conversational spaces—have with clients about climate change and sustainability (related to hair and more broadly). Overall, we found evidence that hairdressers and owners in sustainable salons were able to exert pro-environmental influence over clients, and that this was enhanced by the Mirror Talkers intervention.

Our findings around the social fabric of salons, including trust, client relationships and how salons are a social space, are consistent with existing literature (e.g., Barbour, 2023; Eayrs, 1993; Lukate, 2022). We add to this by highlighting the potential for these spaces and relationships to be used for public engagement with climate change and sustainability. Our findings emphasise the unique skills that hairdressers have for reading and engaging with diverse clients, and developing trusting, safe spaces for holding climate conversations. While researchers can equip people with the tools and knowledge to become better at public engagement, it could be argued that given their daily role in conversing with the public, hairdressers may already have a strong understanding of how to do this successfully. They engage with a wide variety of people daily, are skilled communicators and have much to teach others in terms of reading people and adapting conversations accordingly. Their expertise is also valuable as it is developed through natural, everyday interactions, with an understanding of how to engage with people in constantly evolving, real life situations. They maintain trusting and long-term relationships with the public, sometimes over decades, and are also trusted in climate change and sustainability conversations.

Climate and sustainability conversations are already happening in sustainable salons. The results show that hairdressers are aware of the need to tailor discussions and understand clients, reflecting what is known from public engagement research, for example, about framing (Badullovich, Grant and Colvin, 2020) and values (Climate Outreach, 2024a). Previous research revealed several ways of framing climate action that consistently resonate with the British public (Climate Outreach, 2024a). Some of these were evident in client conversations which sometimes began with haircare as a point of connection, but expanded to broader conversations about energy, transport, food, investments, carbon literacy and intergenerational responsibility. These framings include community and collective action as opportunities to build up local areas and strengthen social connection, discussing positive individual actions as a prompt for collective action and positive impacts on health and wellbeing (ibid). Key principles of everyday climate conversations (Webster and Marshall, 2019) are already embedded in client interactions, with salon owners/directors speaking about finding common ground, describing their own sustainability or climate journeys, taking action, and listening and learning from clients. This includes understanding when clients may prefer not to discuss these topics.

Our findings show that for the hair salons we engaged with, climate and sustainability conversations are also somewhat influencing the mindset and behaviour of clients. While this may seem obvious as our studies primarily engaged with sustainable salons, it was not known if or how salon actions translate to client conversations and actions. Physical items acted as prompts for conversations in both studies. Sustainable hair care conversations were successfully prompted by the Mirror Talkers stickers and viewed positively by clients in terms of enjoyment and learning. Hairdressers tended to underestimate their value with almost half reporting that they did not know what impact such conversations would have, although this could be due to trialling something novel. In fact, almost three-quarters of clients said they were likely to change their hair practices following the Mirror Talkers conversations. These findings support research which highlights the efficacy of leading by example in public engagement on climate change (Westlake et al., 2024). Also, while taking climate action was seen by a small number of hairdressers and clients as being an “eco warrior” or “activist”, there are many ways to be a climate activist, including as an influencer through collaborating with people at an individual scale (Kirsop-Taylor et al., 2023). It may be that hairdressers have not yet envisaged what a climate activist could look like in their profession and how this can differ from high profile activists they might see in the media.

Hairdressing is a widespread industry and has considerable untapped potential regarding the influence that hairdressers could have on clients. While the members of the Green Salon Collective participating in this study are strongly engaged with climate and sustainability and understand the impact they can have, they represent a small minority of the sector. Both studies demonstrated the importance of dialogue-based approaches as well as one-way information provision, or through physically showcasing items. In other words, being vocal and visible around climate change and sustainability. Many salon owners/directors thought they could have an influence on clients, and were receptive to support and interventions such as Mirror Talkers.

In exploring the circular economy in the hairdressing industry, Hodgson et al. (2024, p.512) call for “thoughtful consideration of diverse pathways to achieve sustainability”, emphasising that sustainability can be approached through multiple, often underexplored routes. Our studies show that hairdressers themselves represent one such pathway. This has implications for climate policy for the industrial and commercial sector. While larger businesses attract most attention from policymakers, SMEs including salons represent 50% of business emissions (Department for Business, Energy and Industrial Strategy, 2019). Policy approaches which do target SMEs have tended to focus on technical building audits and energy efficiency measures (Hampton et al., 2023). Our findings highlight the role of salons as local hubs for social connection, and their potential for helping to overcome pluralistic ignorance relating to climate action. Other enterprises such as cafés and restaurants, barbershops and beauty salons, and farmers markets may serve similar functions. Supporting and enhancing these SMEs’ capabilities for social influence on climate action demands a new approach to local enterprise policy, one that is people-centric and focused on everyday interactions, rather than only technical strategies.

There are also implications for education and skills policy. Although many hairdressers have considerable knowledge and experience engaging with clients, there is potential for training and development around climate change and sustainability. They need support both to make their businesses more sustainable and to better use their position to influence clients. In UK-wide interviews, we asked what it would take for more hairdressers and salons to engage with their clients on climate change and sustainability. Almost all wanted more education and knowledge. Baden (University of Southampton, 2019) has already created a sustainable salon certification scheme and helped to embed sustainability practices into the occupational standards for hairdressing and beauty therapy, which may help to build confidence and skills amongst the next generation of hairdressers. However, our findings demonstrate the importance of climate conversations for promoting pro-environmental behaviour and raising the profile of climate change amongst broad publics, and there is potential for incorporating client sustainability advice into education and training programmes. The success of the Mirror Talkers project also demonstrates the potential for low-cost interventions to help highlight salons’ sustainability efforts and initiate climate conversations. There is potential for a wider roll-out of this scheme, and for similar interventions in other settings.

## Conclusion

Our research contributes to the literature on public engagement and climate change, highlighting the importance of everyday social influence. Focusing on the unique profession of hairdressers and the salon as a space for public engagement, we highlight the importance of *everyday influencers* in shaping perceptions and behaviours related to sustainability and climate action. Our studies explore dialogue-based approaches to empower hairdressers and engage the public. We respond to calls by Ettinger and Painter (2023) to gather evidence on ‘real-world’ conversations, doing so by attending to the ways in which trust, knowledge-exchange and influence emerge in the hairdresser-client relationship.

A limitation of the Mirror Talkers study is a small sample size for the interviews. Some closed questions were included in interviews, as these are easier for time-limited participants. However, this format offered less scope for in-depth qualitative analysis. In addition, the results may not be generalisable to salons that have not done the sustainable salon certification. For GoZero, while salon owners and directors were able to speak about their many years of experience engaging with clients, no interviews were held with clients themselves. Therefore, it was not possible to hear directly from clients if and how they changed their behaviour.

There are many avenues for further research on the role of everyday influencers for climate action, and hairdressers specifically. First, there is scope to gather more evidence on the client perspective. While we surveyed clients involved in the Mirror Talkers intervention, there is a need to understand longer-term impacts of climate conversations on haircare practices and other environmental behaviours. This includes evaluating the effects of further interventions. Second, these studies focused on salons—catering largely to women—due to the opportunity afforded by GSC network. There is a need to compare and contrast the influence of barbers and barbershops as potential spaces for climate conversations. Also, given the lack of an equivalent sustainability initiative, there is a need for transdisciplinary collaborations to apply lessons from the initiatives we have reported on. Thirdly, hairdressing is a significant economic activity around the world—research is needed on their everyday influence for climate action outside the UK. Lastly, future research may contrast the findings of this paper with the *actual* and *potential for* influence amongst other enterprises, professions, and the conditions for creating spaces of trust and learning, for productive climate conversations.

Public engagement on climate change is vital to build broad support for climate policy, yet governments frequently underestimate its importance. Deliberative approaches such as citizens assemblies and e-petitions have become more widespread, and public information campaigns are sporadically deployed to raise awareness. Yielding the power of everyday influencers to build public consensus is an under-utilised strategy which demands new approaches to climate policy. These include recognising and empowering professionals such as hairdressers with education, skills and resources to fulfil their potential.

## Supplementary information

Supplementary Information

## Data Availability

The datasets generated during the current studies are not publicly available due to the risk of individual identification but are available from the corresponding author on reasonable request.
