# Supplementary material for: Public engagement and climate change: exploring the role of hairdressers as everyday influencers
Source: Humanit Soc Sci Commun. 2026 Feb 26;13(1):415. doi: 10.1057/s41599-026-06781-4 (PMC13056535; doi:10.1057/s41599-026-06781-4)
Supplement: Supplementary file 1 — Supplementary Information [file 41599_2026_6781_MOESM1_ESM.docx]

Supplementary data

Contents

**GoZero**
[Interview protocol 2](#_Toc188535072)

**Mirror Talkers**
[Mirror Talkers sticker designs 5](#_Toc188535073)

[Survey questions 11](#_Toc188535074)

[Hairdresser survey 11](#_Toc188535075)

[Salon owner survey 13](#_Toc188535076)

[Client survey 16](#_Toc188535077)

[Interview protocol 18](#_Toc188535078)

GoZero

All interview questions were newly created apart from Question 9 which was adapted from [Britain Talks Climate](https://climateoutreach.org/britain-talks-climate/previous-research/trust/).

# Interview protocol

Section 1: Background context

1. **Could you give me a brief 1-minute overview of what actions your business is currently taking to address climate change or broader sustainability issues?**

*Prompts:*

*- Climate change = energy use, travel, food/drink*

*- Sustainability = water, waste*

Section 2: Client influence

I want to ask some questions about your engagement with clients.

1. **The Green Salon Collective has a ‘Green Bible’ which is a guide to running a sustainable and ethical hair salon. In the guide it has a recommendation to: *“Tell customers about all the incredible things you’re doing as a salon to help planet and people”*. Do you engage with your clients about the climate change and sustainability actions that your salon is taking?**
2. [If yes to Q2] **In what ways?**

*Prompts: What (topics), when (on site, before/after), how (discussion i.e. two-way interaction or via business materials e.g. menu/information, general communications i.e. one-way interaction)*

1. [If no to Q2] **Why not?**
2. **Do you engage with your clients about climate change and sustainability as a *general topic* of conversation? So not about what the salon is doing.**

*Prompts: Does it come up in conversation? Who raises it?*

1. [If yes to above questions] **When you’re engaging with clients on these topics, do you think you’re influencing them? (or could influence them)**

*Prompts: The way clients think about these issues, or their behaviour. What is it about how you’re engaging that you think influences them? E.g. conversation*

1. **Do you have any examples of when you felt you’ve been able to influence clients on climate change or sustainability?**
2. **Have there been any examples of when you have you been influenced by clients?**
3. [If they DO speak to clients] **What response do you get from clients when you talk about these topics?**

*Prompts:*

*- Easy/difficult to engage and why?*

*- [If hard] What would help you overcome this?*

*- Do you let clients lead the topics of conversation (i.e. only react if the topic comes up)?*

*- Particular topics that are easier or harder to talk about?*

1. **How much do you think clients trust you (in your role as a hairdresser) to talk about climate change and sustainability?**

*Prompts: Do they value what you have to say? Do they listen to you/are they interested? Do they see you as a trusted source of information?*

1. **How do you decide which clients to engage with – are there types of clients that are easier to engage with than others?**

*Prompt: Demographics or personality traits*

1. [If they DON’T speak to clients] **Is there anything stopping you from engaging with clients on these topics?**

*Prompts: Don’t want to preach? Shy? Lack of knowledge? Think clients won’t want to hear it?*

1. **What would it take for more hairdressers and hair salons to engage with their clients on climate change and sustainability?**

*Prompts: any support or guidance? e.g. from industry, suppliers, government/policymakers*

1. **Do you pass the green fee on to clients?**

Section 3: Wider influence

We’ve spoken about clients but now I’d like to ask a final question about your potential influence more broadly.

1. **Are there any other ways that you think you as a hairdresser or your hair salon have influence around climate change and sustainability? This could be your peers and the wider sector (other salons, networks) or upwards influence (suppliers, government, decision makers)**

*Prompts: Anyone else you’re interacting with in your role as a hairdresser*

Section 4: Closing questions

1. **Do you have anything else to add that we haven’t spoken about in terms of the influence that hairdressers have on climate change and sustainability?**

Mirror Talkers

# Mirror Talkers sticker designs

All Mirror Talkers stickers are publicly available on the [Green Salon Collective website](https://www.greensaloncollective.com/collections/marketing-clothes-stickers-accessories).

**Supplementary Figure F1. Mirror Talkers sticker design 1.**


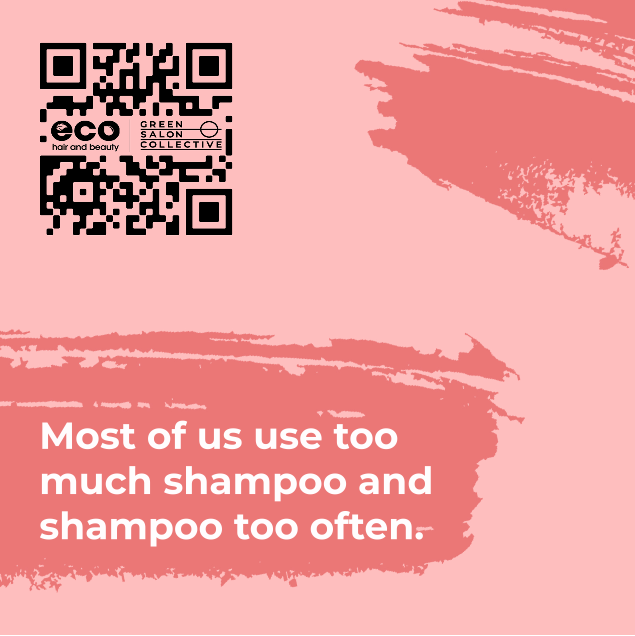


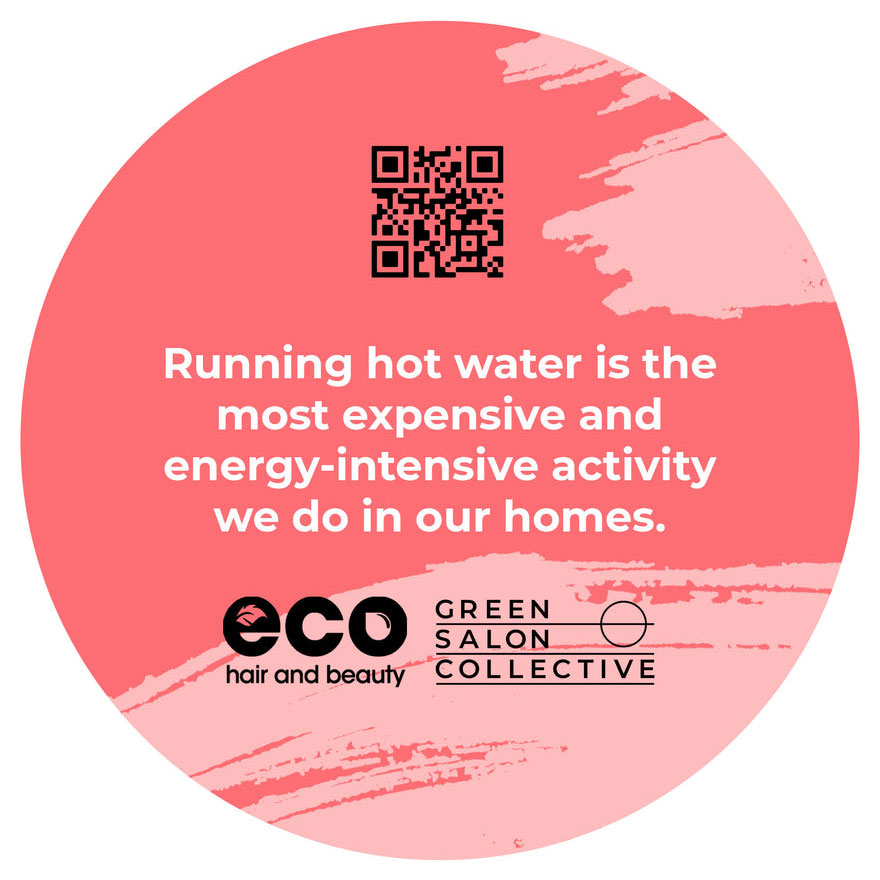
 **Supplementary Figure F2. Mirror Talkers sticker design 2.**

**Supplementary Figure F3. Mirror Talkers sticker design 3.**


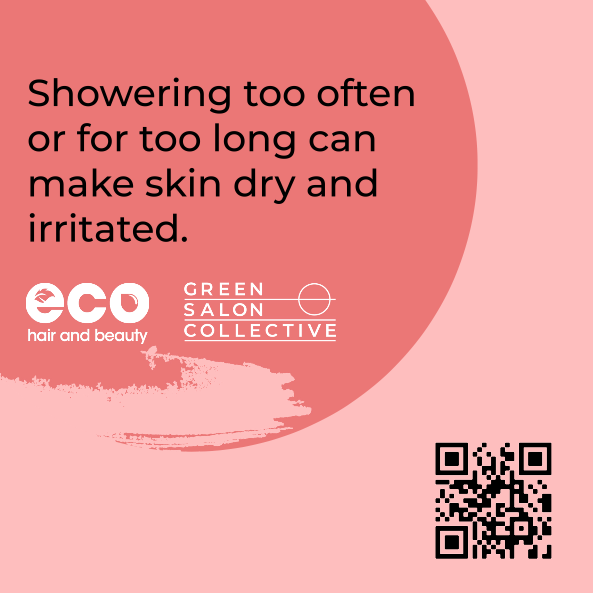


**Supplementary**
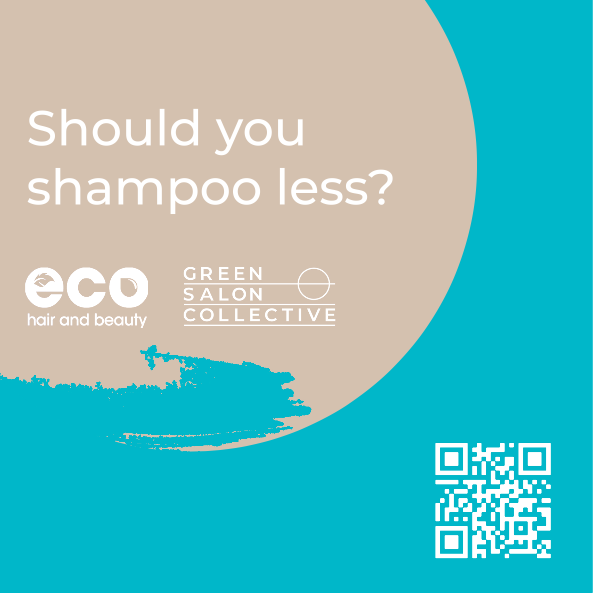
**Figure F4. Mirror Talkers sticker design 4.**

**Supplementary Figure F5. Mirror Talkers sticker design 5.**


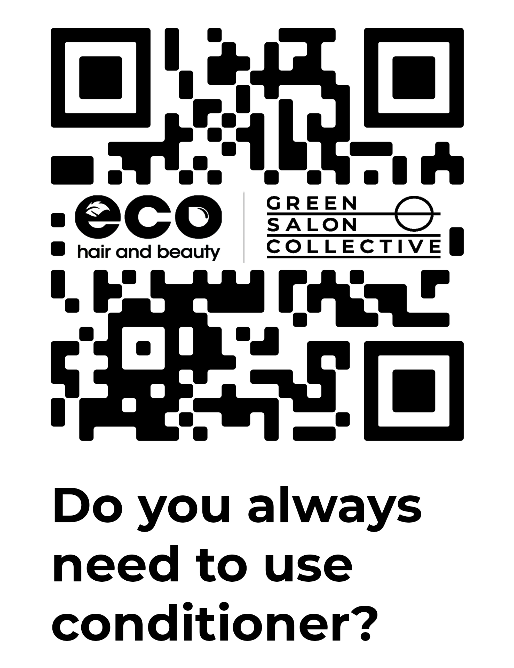


**Supplementary Figure F6. Mirror Talkers sticker design 6.**


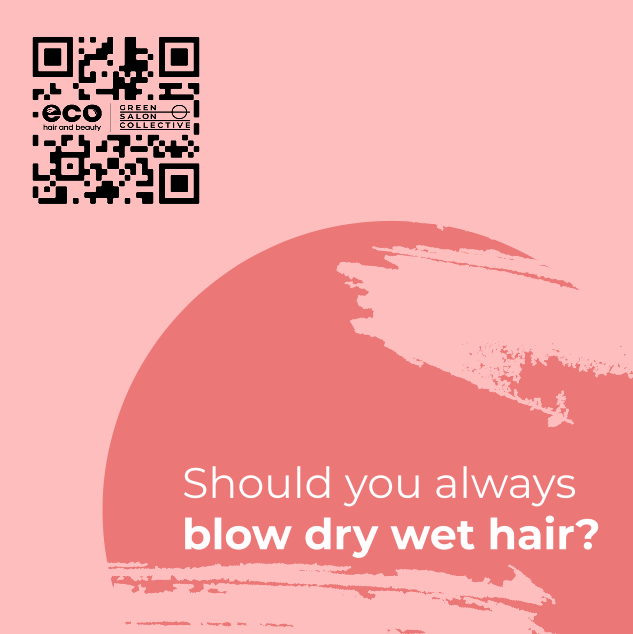


**Supplementary Figure F7. Mirror Talkers sticker design 7.**


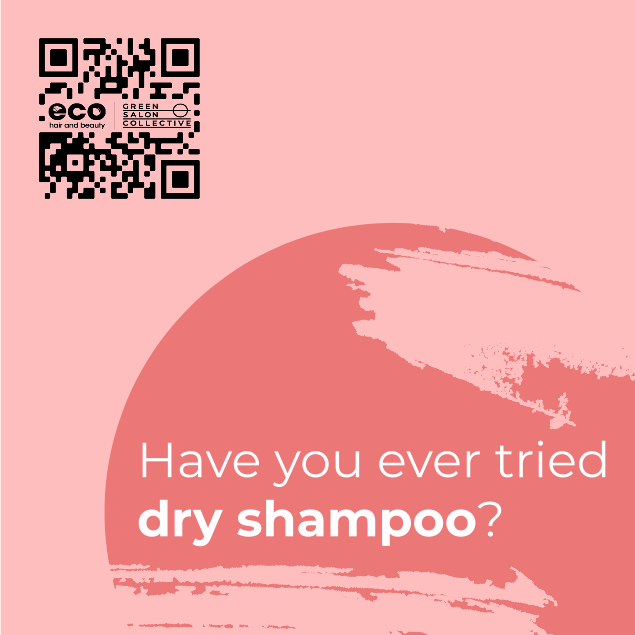


**Supplementary**
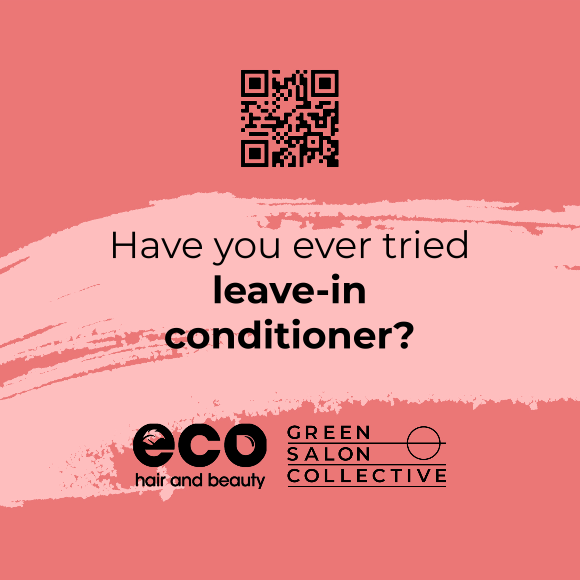
**Figure F8. Mirror Talkers sticker design 8.**

**Supplementary Figure F9. Mirror Talkers sticker design 9.**


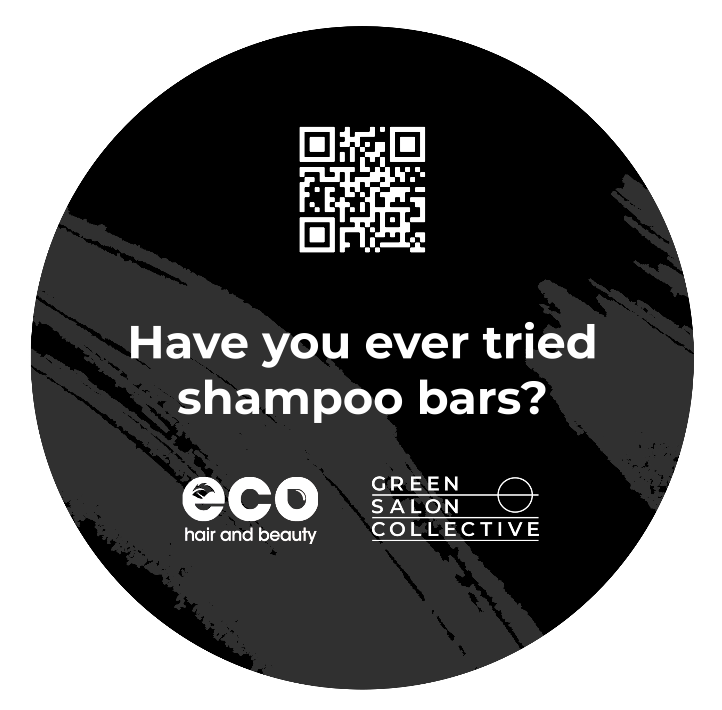


**Supplementary**
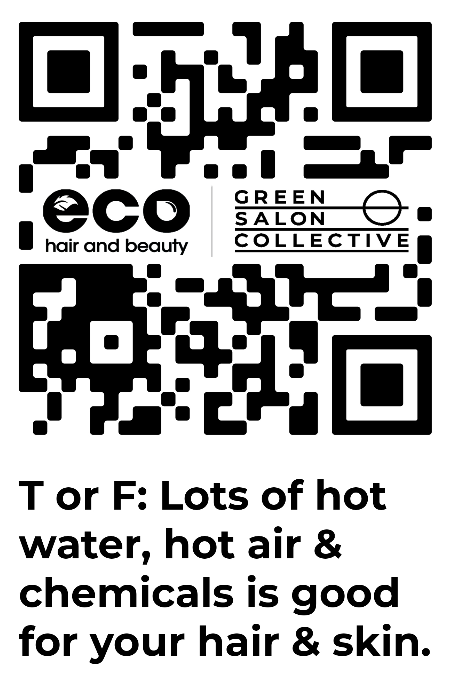
**Figure F10. Mirror Talkers sticker design 10.**

**Supplementary Figure F11. Mirror Talkers sticker design 11.**

**
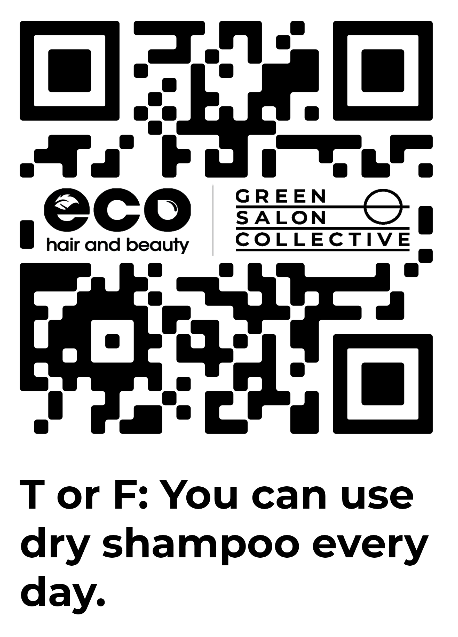
**

**Supplementary
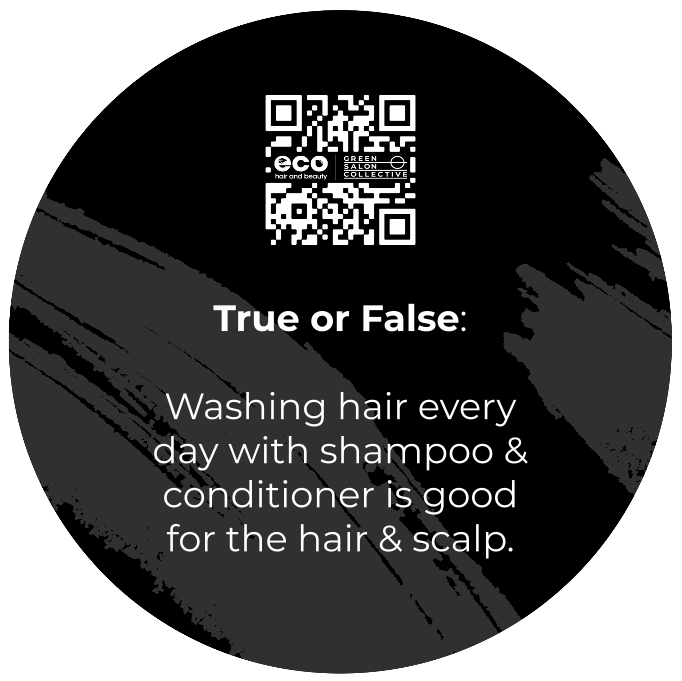
Figure F12. Mirror Talkers sticker design 12.**

# Survey questions

## Hairdresser survey

1. **Tick if you agree for your data to be used for the purpose of this study.**
   1. Yes, I agree.
2. **Which Mirror Talkers did you work with?** [Multiple choice, multiple answer]
   1. Most of us use too much shampoo and shampoo too often.
   2. Running hot water is the most expensive and energy-intensive activity we do in our homes.
   3. Showering too often or for too long can make skin dry and irritated.
   4. Should you shampoo less?
   5. Do you always need to use conditioner?
   6. Should you always blow dry wet hair?
   7. Have you ever tried dry shampoo?
   8. Have you ever tried leave-in conditioner?
   9. Have you ever tried shampoo bars?
   10. T or F: Lots of hot water, hot air and chemicals is good for your hair and skin.
   11. T or F: You can use dry shampoo every day.
   12. T or F: Washing hair everyday with shampoo and conditioner is good for the hair and scalp.
3. **How helpful was the training information for those Mirror Talkers?** [1 = Not helpful, 5 = Very helpful]
   1. 1
   2. 2
   3. 3
   4. 4
   5. 5
4. **How effective were the Mirror Talkers at prompting conversations around sustainable haircare practices?** [1 = Not effective, 5 = Very effective]
   1. 1
   2. 2
   3. 3
   4. 4
   5. 5
5. **How did those conversations go? (Select all that apply)** [Multiple choice, multiple answer]
   1. They were enjoyable.
   2. They were fine.
   3. They were uncomfortable.
   4. I felt confident in what I was saying.
   5. I didn't feel confident in what I was saying.
   6. Other: [open text box]
6. **Do you believe those conversations will have a positive effect on your clients' haircare routines?** [Multiple choice, single answer]
   1. Yes
   2. No
   3. I don't know
   4. Other: [open text box]
7. **If you used more than one Mirror Talker, which one gave rise to the most productive conversations around sustainable haircare?** [Multiple choice, single answer]
   1. Most of us use too much shampoo and shampoo too often.
   2. Running hot water is the most expensive and energy-intensive activity we do in our homes.
   3. Showering too often or for too long can make skin dry and irritated.
   4. Should you shampoo less?
   5. Do you always need to use conditioner?
   6. Should you always blow dry wet hair?
   7. Have you ever tried dry shampoo?
   8. Have you ever tried leave-in conditioner?
   9. Have you ever tried shampoo bars?
   10. T or F: Lots of hot water, hot air and chemicals is good for your hair and skin.
   11. T or F: You can use dry shampoo every day.
   12. T or F: Washing hair everyday with shampoo and conditioner is good for the hair and scalp.
   13. None of them did.
8. **We will be conducting one-to-one interviews with hairdressers to better understand how effective they feel Mirror Talkers are at prompting conversations around sustainable haircare practices and to give them the opportunity to suggest ways to improve their use. If you are interested in being interviewed, please provide your name and email address here.**
9. [open text box]
10. **TO ENTER INTO OUR PRIZE DRAW please type the name of your salon here. We will not correlate this information with the rest of the data. It will only be used to select winners and then the information will be deleted.**
    1. [open text box]

## Salon owner survey

1. **Tick if you agree for your data to be used for the purpose of this study.**
2. Yes, I agree.
3. **Which Mirror Talkers did you work with?** [Multiple choice, multiple answer]
4. Most of us use too much shampoo and shampoo too often.
   1. Running hot water is the most expensive and energy-intensive activity we do in our homes.
   2. Showering too often or for too long can make skin dry and irritated.
   3. Should you shampoo less?
   4. Do you always need to use conditioner?
   5. Should you always blow dry wet hair?
   6. Have you ever tried dry shampoo?
   7. Have you ever tried leave-in conditioner?
   8. Have you ever tried shampoo bars?
   9. T or F: Lots of hot water, hot air and chemicals is good for your hair and skin.
   10. T or F: You can use dry shampoo every day.
   11. T or F: Washing hair everyday with shampoo and conditioner is good for the hair and scalp.
5. **Why did you choose to work with these Eco Tips? Feel free to explain why, if you wish.**
   1. [open text box]
6. **How helpful was the training information for those Mirror Talkers?** [1 = Not helpful, 5 = Very helpful]
   1. 1
   2. 2
   3. 3
   4. 4
   5. 5
7. **Did you participate in the live online training sessions? Feel free to explain why or why not, if you wish.**
   1. [open text box]
8. **Did you watch the training videos? Feel free to explain why or why not, if you wish.**
   1. [open text box]
9. **Did you read all of the material in the QR links corresponding to your chosen Eco Tips? Feel free to explain why or why not, if you wish.**
   1. [open text box]
10. **How effective were the Mirror Talkers at prompting conversations around sustainable haircare practices?** [1 = Not effective, 5 = Very effective]
    1. 1
    2. 2
    3. 3
    4. 4
    5. 5
11. **Please explain your answer in your own words here, if you wish. (How effective were the Mirror Talkers...)**
    1. [open text box]
12. **How did those conversations go? (Select all that apply)** [Multiple choice, multiple answer]
    1. They were enjoyable.
    2. They were fine.
    3. They were uncomfortable.
    4. I felt confident in what I was saying.
    5. I didn't feel confident in what I was saying.
    6. Other: [open text box]
13. **Please explain your answer in your own words here, if you wish. (How did these conversations go?)**
    1. [open text box]
14. **Do you believe those conversations will have a positive effect on your clients' haircare routines?** [Multiple choice, single answer]
    1. Yes
    2. No
    3. I don't know
    4. Other: [open text box]
15. **Please explain your answer in your own words here, if you wish. (Do you believe those conversations will have...?)**
    1. [open text box]
16. **If you used more than one Mirror Talker, which one gave rise to the most productive conversations around sustainable haircare?** [Multiple choice, single answer]
    1. Most of us use too much shampoo and shampoo too often.
    2. Running hot water is the most expensive and energy-intensive activity we do in our homes.
    3. Showering too often or for too long can make skin dry and irritated.
    4. Should you shampoo less?
    5. Do you always need to use conditioner?
    6. Should you always blow dry wet hair?
    7. Have you ever tried dry shampoo?
    8. Have you ever tried leave-in conditioner?
    9. Have you ever tried shampoo bars?
    10. T or F: Lots of hot water, hot air and chemicals is good for your hair and skin.
    11. T or F: You can use dry shampoo every day.
    12. T or F: Washing hair everyday with shampoo and conditioner is good for the hair and scalp.
    13. None of them did.
17. **Please explain your answer in your own words here, if you wish. (Which Mirror Talker(s) gave rise to...?)**
    1. [open text box]
18. **We are hosting an evening event in late November to share our findings and record mini interviews with our participants. What day of the week would suit you best for such an occasion?** [Multiple choice, multiple answer]
    1. Monday
    2. Tuesday
    3. Wednesday
    4. Thursday
    5. Friday
    6. Saturday
    7. Sunday
19. **We will be conducting one-to-one interviews with salon owners to better understand how effective they feel Mirror Talkers are at prompting conversations around sustainable haircare practices and to give them the opportunity to suggest ways to improve their use. If you are interested in being interviewed, please provide your name and email address here. (If you have already submitted your details for this in the Hairdresser Survey, please add them again here.)**
    1. [open text box]

## Client survey

1. **Tick if you agree for your data to be used for the purpose of this study.**
   1. Yes, I agree.
2. **Which Mirror Talkers did you work with?** [Multiple choice, multiple answer]
   1. Most of us use too much shampoo and shampoo too often.
   2. Running hot water is the most expensive and energy-intensive activity we do in our homes.
   3. Showering too often or for too long can make skin dry and irritated.
   4. Should you shampoo less?
   5. Do you always need to use conditioner?
   6. Should you always blow dry wet hair?
   7. Have you ever tried dry shampoo?
   8. Have you ever tried leave-in conditioner?
   9. Have you ever tried shampoo bars?
   10. T or F: Lots of hot water, hot air and chemicals is good for your hair and skin.
   11. T or F: You can use dry shampoo every day.
   12. T or F: Washing hair everyday with shampoo and conditioner is good for the hair and scalp.
3. **Did you discuss the Mirror Talker with your hairdresser?** [Multiple choice, single answer]
   1. Yes
   2. No
4. **If “yes”, tell me how that went. (Select all that apply)** [Multiple choice, multiple answer]
   1. The chat was enjoyable.
   2. The chat was fine.
   3. The chat was uncomfortable.
   4. I learned something new.
   5. I didn’t learn anything new.
   6. Other: [open text box]
5. **Why did you scan the QR code?** [Multiple choice, single answer]
   1. I was curious.
   2. I was prompted by the hairdresser.
   3. I wanted to learn more.
   4. I don’t know.
   5. Other: [open text box]
6. **Based on this experience, how likely is it that you will change your haircare routine?** [1 = Not likely, 5 = Very likely]
   1. 1
   2. 2
   3. 3
   4. 4
   5. 5
7. **We will be conducting one-to-one interviews with clients who experienced our Mirror Talkers to better understand how effective they are at prompting conversations around sustainable haircare practices. If you are interested in being interviewed, please provide your name and email address here.**
   1. [open text box]

# Interview protocol

1. **Can I have your permission to post about this interview on Green Salon Collective (GSC) social media, take a screenshot for that purpose, and record the interview for research purposes?**
2. **Could you please introduce yourself by stating your name and the name of the salon you own/work in/visit?**
3. **Do you have any questions about the Mirror Talkers study beyond what I have just explained to you?**
4. **The eco tip you were presented with (e.g., about the effects of hot water, hot air, and chemicals on hair and skin) – was this something you were familiar with before encountering it on the Mirror Talker?**
5. **How did your conversation with your hairdresser/client on this topic unfold? What did that interaction look like?**
6. **Hairdressers were provided with background information on health & beauty, money-saving tips, and environmental benefits to help guide these conversations. Did any of these aspects stand out more during your discussion with the hairdresser/client?**
7. **Which of these factors – health, financial savings, or environmental benefits – would you find/do you believe your client would find most compelling for adopting a more sustainable haircare routine?**
8. **Did you scan the QR code on the Mirror Talker? If so, did you learn anything further, and was the information easy to understand?**
9. **Since completing the initial survey, have you made/ do you believe your client has made any changes to your/their haircare routine as a result of this experience?**
10. **What are your thoughts on the concept of Mirror Talkers as a way to engage people/ clients on climate-related topics in a practical and approachable way?**
